# What works in advocating for food advertising policy change across an english region – a realist evaluation

**DOI:** 10.1186/s12889-023-16829-8

**Published:** 2023-10-02

**Authors:** Susie Sykes, Megan Watkins, Matthew Bond, Catherine Jenkins, Jane Wills

**Affiliations:** London South Bank University, 101 Borough Road, SE1 0AA London, England

**Keywords:** HFSS advertising, Public health advocacy, Public health policy, Realist evaluation

## Abstract

**Background:**

With increasing recognition of the role of commercial determinants of health, local areas in England have sought to restrict the advertising of products high in fat, salt and sugar (HFSS) on council-owned spaces, as part of wider strategies to reduce obesity. While there is some evidence of the impact of such policy change on behaviour, little is known about what works in the process of implementing this policy change.

**Methods:**

Guided by a realist evaluation framework that explores the interaction between context, mechanism and outcomes, this study aims to investigate the factors that influence the restriction of outdoor advertising of HFSS products in one region in England. It refines a programme theory co-produced with stakeholders from 14 local authorities within a region and uses multiple data sources from each area with an in-depth examination of four case study sites. Data sources include longitudinal realist interviews, focus groups and surveys with policy advocates and policy stakeholders. Data were analysed retroductively to understand the causal link between context, mechanism and outcomes.

**Results:**

Outcomes were driven by five dominant mechanisms: a strategic and staggered approach to stakeholder engagement, gathering intelligence, identifying policy champions, building relationships, reframing the issue; and two secondary mechanisms of amplifying the issue and increasing public will. These led to varied outcomes with no changes in formal policy position within the evaluation period but draft policy guidance in place and changes in political will demonstrated. Dominant context factors influencing change included having a named and resourced policy advocate in place supported by an external Community of Improvement and having existing aligned local objectives. Organisational complexity and change, financial concerns, lack of local examples, ideological positions and the pandemic were also influencing contextual factors.

**Conclusion:**

Effecting policy change in this area requires the commitment of an extended period and the valuing of short-term policy outcomes, such as increasing political will. The importance of a resourced and well-supported policy advocate to lead this work is fundamental and the commercially sensitive nature of this policy change means that a complex interplay of mechanisms is required which may be dominated by a strategically staggered approach to stakeholder engagement.

## Background

Increasing attention has been placed on the role of commercial determinants of health of obesity [1] including the marketing of less healthy foods such as those high in fat, salt and sugar (HFSS). This is particularly influential on children and adolescents [2–4] impacting awareness, attitudes and consumption [5, 6]. Exposure to advertising of unhealthy food and drinks has been shown to disproportionately affect those from deprived or ethnic minority backgrounds [7] and while regulations exist in the UK around television advertising of HFSS foods targeted at children, no national regulations exist for other advertising spaces such as out of home or outdoor advertising that include billboards, bus shelters and digital displays. These forms of advertising are pervasive, reaching 98% of the population each week and generating significant income (510 million GBP annually) [8].

Local initiatives in England, have sought to restrict the advertising of HFSS products on public transport and on outdoor spaces. In 2019, for example, the Greater London Authority introduced restrictions on the advertising of HFSS products on Transport for London (TfL), for which an evaluation showed an association between the restrictions and reductions in energy, sugar and fat purchased from HFSS products [9]. Modelling suggests that three years post-intervention there will be (i) 94,867 fewer people in London with obesity, (ii) a reduction in the incidence of diabetes and cardiovascular disease by 2,857 and 1,915 cases respectively, (iii) production of approximately 16,394 additional quality-adjusted life years and (iv) a saving of £218 million in NHS and social care costs with greater benefits to socioeconomically deprived groups [10]. Little is known about the process of implementing such a policy change to restrict outdoor HFSS advertising, including how such restrictions should be designed, monitored, enforced, maintained and adapted. A process evaluation of the TfL restrictions demonstrated both practical and political challenges during the development and implementation of the policy [11]. London may however, represent a unique context for policy change as TfL is a government body responsible for London’s transport system and is governed by the Mayor of London with different regulatory powers to regions outside London which may comprise several different and complex local government structures.

The process of trying to effect policy change is known as advocacy. Advocacy is a core function of public health and policy advocates, who may operate at a specialist or practitioner level, and is a process of seeking to influence policies or create circumstances that maximise the potential for community health and well-being [12] Influencing public policy change can be difficult and complex and as Clavier and de Leeuw [13] point out it is not linear and involves numerous interactions with a variety of stakeholders. There is a small evidence base examining the policy advocacy process in the wider related field of nutrition policy. This identifies a series of mechanisms required for the achievement of a policy goal in this area. These mechanisms include intelligence gathering, investing in relationships, developing a clear and unified solution, employing a policy entrepreneur, engaging policy champions, increasing public will, re-framing and amplifying the issue [14–16] but is a framework developed for advocacy to influence national government policy rather than policy at a local or regional level. The importance of understanding and engaging with stakeholders (including individuals, groups and organisations that have an interest in or are affected by the policy area) as part of this process as well as the central role played by policy entrepreneurs or advocates is emphasised across the literature [15, 17–19].

This study aims to investigate further the factors that influence the achievement of advocacy goals to restrict outdoor advertising of HFSS products in one region in England. The policy goal included a restriction in the marketing of HFSS products on any advertising generated by the council themselves and advertising by third parties on outdoor council-owned spaces. Fourteen local authorities, each with their own strategies on obesity, came together as a “Healthy Weight and Physical Activity Community of Improvement” (CoI) to implement a regional approach to the development of local policies to support the reduction of exposure to HFSS products. This supports the seventh commitment in the Local Authority Declaration on Healthy Weight produced by Food Active [20] and contributes to the regional work programme to reduce obesity across the region to deliver on the government policy on tackling obesity [21]. The regional CoI is made up of policy advocates leading this work from Unitary Authorities, County Councils and District Councils. As such it represents diverse and complex local governance structures as well as varied demographics and with a range of existing policies in place. Each policy advocate sought to lead policy change in their local authority with support from the CoI and therefore represents a specific advocacy context taking place at a local level with advoactes positioned within the organisation and which sought to make change to a commercially sensitive policy within an organisation. Work began in July 2020 with a light touch audit of the current policy position in each area. Funding was secured to provide additional support from a national food advocacy alliance from October 2020 – March 2021, which was subsequently extended to December 2021.

## Methodology and methods

A realist evaluation was conducted which sought to understand ‘what worked, for whom and in what circumstances’. Realist evaluation [22, 23] is a theory-driven model of evaluation based on the assumption that projects and programmes only work under certain conditions and are heavily influenced by the ways in which different actors respond to them and the decisions and actions made along the way. It focuses on the interaction of three elements: the mechanisms of change, the context within which programmes operate and the outcomes they achieve. The realist methodology achieves this through the development of a theory of change that is then tested and refined in a range of cases that offer different contextual settings or mechanisms for delivery. Typically, a realist evaluation is made up of four stages: theory generation, hypotheses generation through context, mechanism, outcome configurations, observations through data collection and analysis and programme specification [23]. These stages are described below and are in line with recommended reporting standards for realist evaluations [24, 25].

### Stage one and two: theory generation and working hypothesis

In realist evaluations, theory is used to explain the underlying logic of programmes, which is then tested in the evidence through working hypotheses which are based on context-mechanism-outcome configurations [26]. The Theory of Change informing this evaluation was developed through a series of workshops with the CoI. The workshops used logic modelling and allowed CoI participants to draw on their experiences from practice, the contextual factors influencing their work and a scoping review of literature. Given the lack of literature specifically looking at advocacy for restrictions on HFSS advertising, this rapid scoping review looked more widely at the international literature on advocacy as a function of public health and advocacy within the field of nutrition. The final agreed theory of change specifically draws upon a conceptual model developed by Cullerton et al [16] within the context of nutrition advocacy which synthesises policy process and network theories to develop a process for effective policy action and seeks to suggest a relationship between mechanisms and outcomes. The resultant programme theory is represented in a series of if-then statements [27]:

IN:

- a complex regional governance structure with varied ideological positions and resource constraints.

IF policy advocates:

- gather local and national intelligence.
- invest in relationships with policy stakeholders and champions.
- develop a clear and unified policy solution.
- re-frame and amplify the issue.

AND:

- increase public will.

THEN:

- there would be an increase in political will among policy stakeholders

SO THAT

- revised local guidelines and contracts restricting the advertising of HFSS foods via council-owned outdoor spaces can be implemented.

In this context the policy stakeholders refer to those representatives within the council that have some stake in or control over the policy change process. The list of context, mechanism, outcome statements (both complete CMOs and part CMOs) that were generated from the programme theory are available online [28].

### Stage three: observations through data collection

The evaluation tested the programme theory statement in a range of cases that offer different contextual settings and mechanisms for delivery. A mixed methods evaluation design was employed which began in March 2021 (but includes retrospective data from local audits undertaken Summer of 2020) and the completion of summative data was February 2022. Baseline and summative data were collected from policy advocates from each local authority, the CoI leads and the lead from the supporting food advocacy alliance group and enhanced baseline, process and summative data were collected from policy advocates and policy stakeholders in four case study local authority sites. Table 1 shows a breakdown of the data collection strategies and focus of each data source.

**Table 1:** Summary of data collection strategies

Data collection tool	Time point	Sample	Purpose
Survey	Baseline and summative	Lead policy advocate from each area (n14)	Explored work undertaken (mechanisms), local context and policy position (outcome), identification of stakeholders, facilitators and barriers to advocacy (context), attitudes to policy (context).
Realist interview	Baseline, formative and summative	Lead policy advocate from each case study site (n4)	Identification of outcomes and movement towards them, examination of mechanisms in programme theory and those used, impact of context on mechanisms, barriers to implementation and strategies for improvement.
Realist interview	Summative	Community of Improvement leads (n2) and lead from supporting food advocacy alliance organisation (n1)	Identification of outcomes and movement towards them, examination of mechanisms in programme theory and those used, impact of context on mechanisms, barriers to implementation and strategies for improvement.
Realist interview	Summative	Engaged policy stakeholders in each case study site (n14)	Explore achievement of political will (as an outcome) towards policy change at personal and council level, awareness of and views on mechanisms applied, identification of contextual barriers and facilitators
Retrospective survey	Summative	Wider policy stakeholders in each case study site (n25)	Retrospective and current personal political will as an outcome, retrospective and current political will of council as an outcome, degree of perceived influence.

The baseline and summative survey questions were developed by the evaluation team following the co-production workshops with the CoI. Interviews conducted as part of a realist evaluation are theory-driven interviews and seek to explicitly test and refine the initial programme theory statement through an examination of what mechanisms have been applied, the perceived impact of context on mechanisms and the perceived relationship with outcomes achieved. Interviews therefore focussed on: the identification of outcomes and movement towards them (such as achieving political will and policy change); examination of mechanisms in the programme theory and any others used (such as intelligence gathering and building relationships); impact of context on mechanisms (such as the governance structures in the local area, local demographics, ideological position) including barriers to implementation and strategies for improvement. Realist interviews are based on a process called the “learner-teacher” cycle which is where elements of the programme theory statement are placed before the respondent for them to comment on and refine [29]. As is typical within realist evaluation, three phases of interviews were undertaken: theory-gleaning interviews, theory refinement interviews and theory consolidation interviews [30]. Questions in the interview schedule were informed by the RAMESES II realist evaluation reporting standards and set of recommended question types [24, 31] and are available online [32]. All data collection tools were piloted by representatives within one of the local authorities and the evaluation Patient and Public Involvement Panel and revisions made accordingly.

#### Sampling

Several contextual variants across the advocacy projects were identified as important during the co-production workshops by CoI members based on their experience and knowledge of their local area. These included project maturity, rural/urban make-up of the area, complexity of demography, the complexity of local political and organisational structure, the degree to which they had existing partnerships with companies and corporations and competing priorities. To explore the relevance and to refine the programme theory statement across these contextual variables, four case study sites were selected through a mapping exercise during the workshops. All Policy Advocates within the CoI, CoI leads and the lead from the food advocacy alliance organisation were included as part of the sample. Policy Stakeholders within the case study sites were selected in consultation with the local policy advocate. They included engaged stakeholders (those council representatives with an interest in or influence over the policy change who had already been actively engaged in the process) and wider stakeholders (those who were seen as influential in the policy change process but who had not yet been approached as part of the stakeholder engagement process). Stakeholders across both groups typically operated at management, strategic or Director levels or were elected members and were drawn from across Directorates.

The approach to sampling meant the case studies included were varied and a range of different policy advocates and stakeholder perspectives were included. A limitation of the approach was the dependency on each area to identify who relevant stakeholders might be.

#### Analysis

A theoretical coding framework was developed based on the 28 context, mechanism, outcome (CMO) configuration statements drawn from the programme theory statement and developed in stage two [33] and is available online [32]. The configuration of these statements into codes which include one or more of the CMO elements allows data to be organised not only according to its defined context, mechanism and outcome but also deliberately seeks to capture the data where the causal pathways between each is described. Data within realist evaluation is typically analysed using a ‘retroductive approach’ [34] which allows for the use of both inductive and deductive logic as well as the insights of the researcher and is based on the belief that understanding the causal links between context, mechanisms and outcomes are not only achieved using observable evidence but come through theory, expertise and common sense.

All interviews from the first round of interview data were transcribed and organised within NVivo 12 software [33, 35]. To ensure familiarisation, two researchers read each transcript, independently coded two transcripts using the theoretical coding framework and carried out additional inductive free-coding, allowing for configuration statements not included in the initial list but appearing in the data, to be captured. Following this initial analysis, the researchers compared coding to ensure a consistent understanding of codes between researchers and allowing for a refinement of the coding framework. All amendments to the framework were captured and recorded as node memos. Subsequent coding was completed by one researcher and the second researcher coded a random 20% sample, plus any data identified by the first researcher as ambiguous in any way. Following the coding process, the dominant context, mechanism, outcome statements were identified and analysed. These were agreed through theorisation discussions across the research team, where both the amount and strength of data supporting the CMO statements were discussed. These took place between the baseline, formative and summative interviews and at the end of the final round of analysis.

### Stage four: programme Specification

Following the analysis of the data, the theory of change and supporting logic model developed during stage one were reviewed in light of the findings. The dominant CMO configurations were used to form the basis of a revised logic model which is presented and discussed below.

## Results

Findings have been organised according to the outcomes that were observed from the advocacy process including the degree to which political will for policy change was secured and whether any change in policy was implemented, followed by the dominant mechanisms that had been applied during the advocacy process and the dominant contextual factors influencing that change. Throughout these sections threads a key finding that this policy context has unique characteristics because of the associated commercial determinants and that this directly influences the mechanisms employed. The findings represent a synthesis of the whole data set. Having categorised and organised the primary data in this way, the realist evaluation sought to identify the causal pathways and relationships between context mechanism and outcome through the generation of refined CMO statements and an examination of the link between the findings and the proposed programme theory.

### Outcomes

#### Achievement of policy change

At the beginning of the evaluation period one area had a written policy prohibiting the advertising of: *‘Fast food / sugary drinks companies, distributors and products (manufacturers of food that is considered unhealthy).”* (Policy Advocate survey). This included no specific mention of HFSS products or definition of unhealthy. One area had a written policy which was described as “*poorly written and not publicly available”* (Policy Advocate survey). One area had an informal unwritten policy prohibiting the advertising of unhealthy foods whilst all other areas had no policy or approved written guidance.

By the end of the evaluation period, no change had occurred in the formal policy position of any councils. However, six areas had produced draft policy guidance that they were confident would be adopted.

#### Changing political Will

For the majority of the local authorities, the Policy Advocates felt there was some acknowledgement within the council that this is a useful policy but not necessarily a priority.

**Fig. 1:**
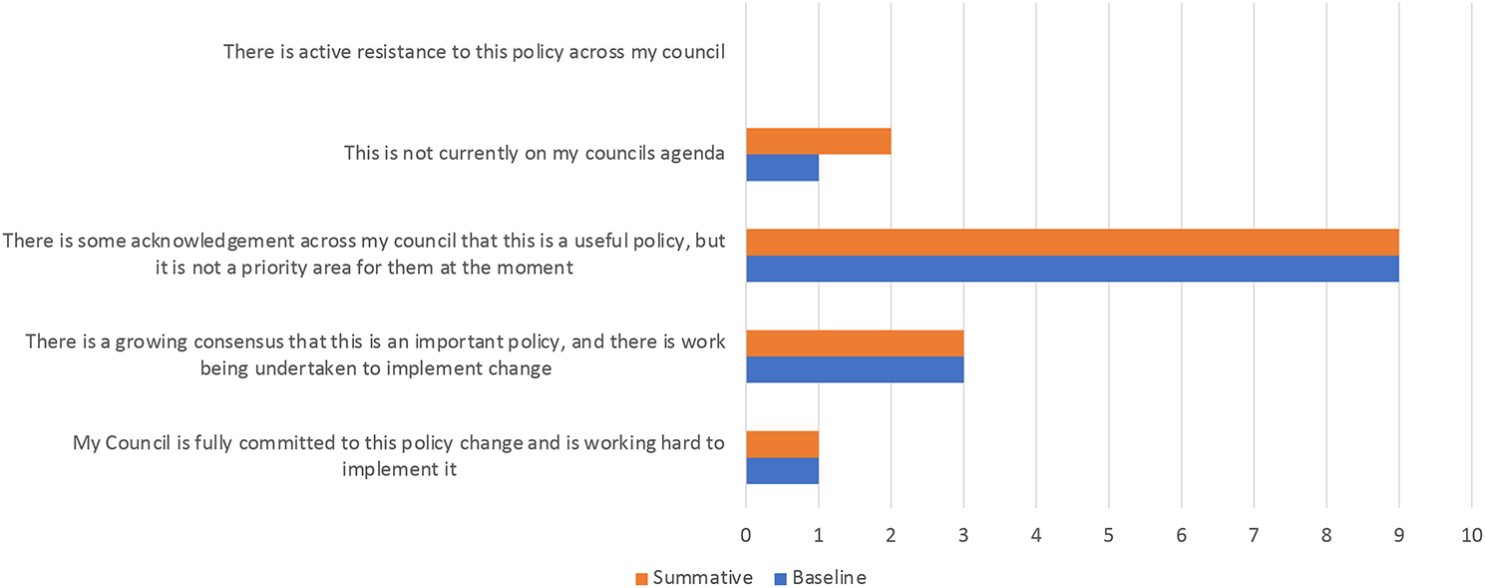
Policy advocates’ perspective of local authority support over time for policy to restrict local advertising of HFSS products on council-owned spaces

This commitment was not seen as having changed over the evaluation period (see Fig. 1.)

**Fig. 2:**
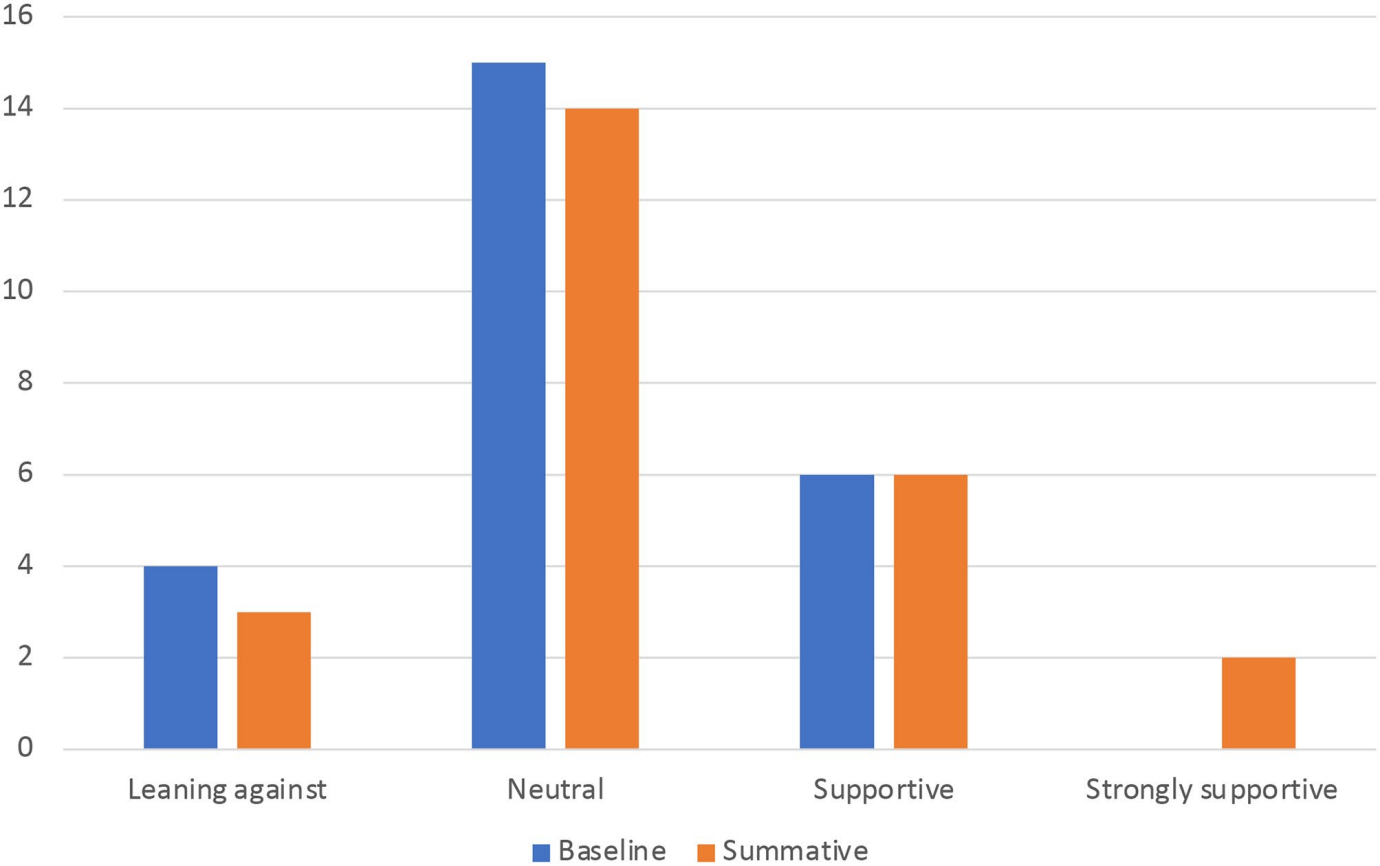
Stakeholder perceptions over time of local authority support for policies to restrict advertising of HFSS products on council-owned spaces

**Fig. 3:**
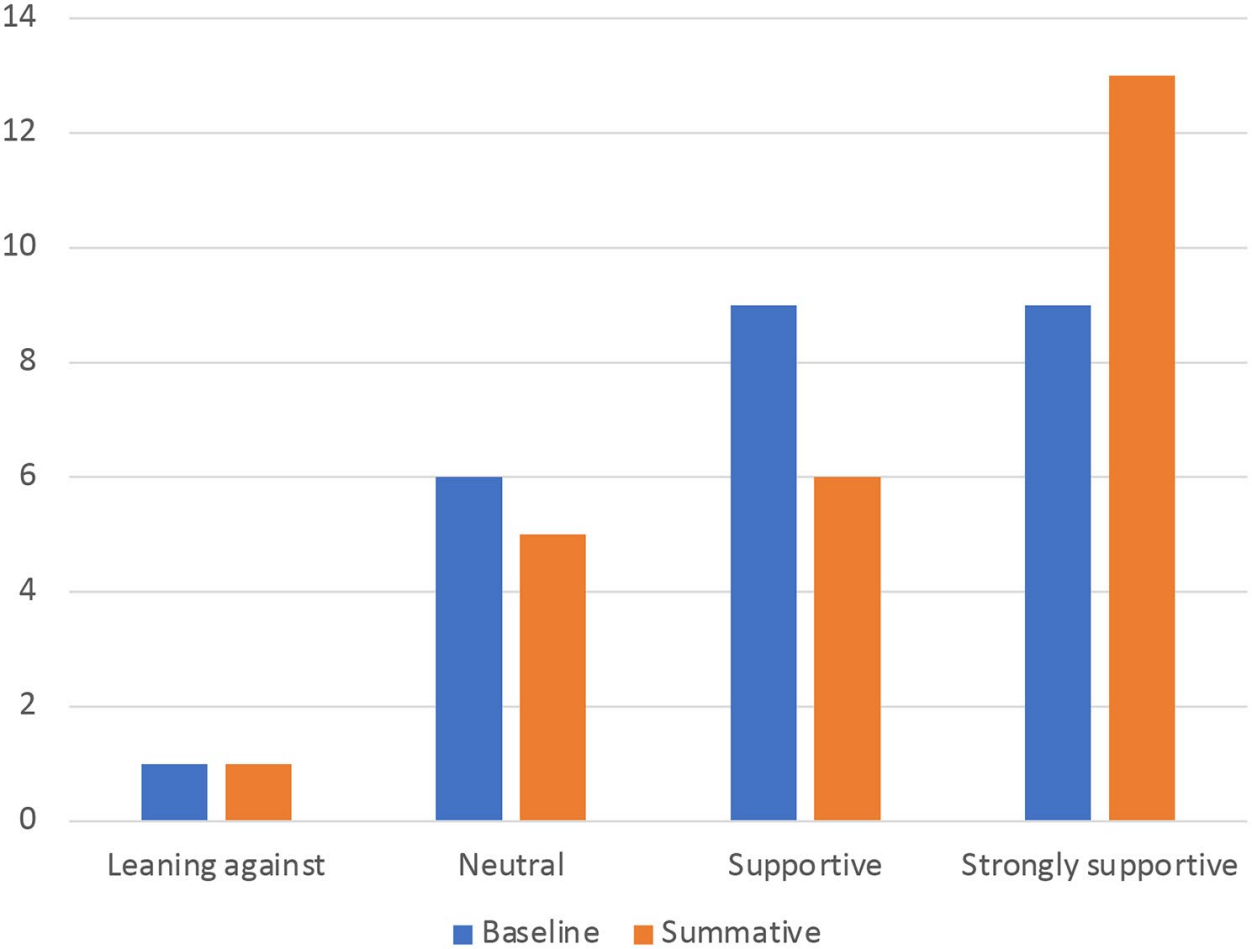
Support over time from wider stakeholders to restrict advertising of HFSS products on council-owned spaces

Data from wider stakeholders in case study sites also reflected this. Their perception was that only a marginal increase in local authority support for the policy had occurred (see Fig. 2):

There was however a slight increase in the number of wider stakeholders who were strongly supportive of the policy themselves (see Fig. 3).

The stakeholders who had been actively engaged by the policy advocates were very supportive of the restricted advertising policy position although several situated their views as personal and separate from their role and the council.

*“I’m a planner, so it’s difficult to say, because planning don’t have any kind of regard for what the advertisement says, so it’s difficult from a planning point of view….From a personal opinion I don’t think we should advertise them at all”* (SH11).

### Mechanisms for policy change

Policy advocates engaged in several mechanisms to effect policy change. These included employing a strategic and staggered approach to stakeholder engagement, identifying policy champions, gathering intelligence, building relationships and reframing the issue. Mechanisms often employed within policy advocacy but not widely adopted in this work include amplifying the issue and increasing public will.

#### A strategic and staggered stakeholder engagement

The most dominant mechanism informing the work of the Policy Advocates was a strategic and staggered stakeholder engagement approach. This involved securing support from senior leadership/management teams and then sequentially across the council, before wider, external engagement. During this process, wider and open discussion about the work was deliberately kept to a minimum. This was driven by a desire to manage fear and potential resistance to the policy change, to ensure a full case was prepared and to manage the potential risk of counter-lobbying by powerful external industry bodies. The drivers of this mechanism can be seen to be unique to advocacy taking place within a commercial context.

*“We are being very careful about who knows about this project at this stage, due to the risk of lobbying by providers of food HFSS”* (Policy Advocate, Survey).

The sequencing and speed of engagement differed across areas and decision-making was undertaken pragmatically, informally and in consultation with the external support structures provided by the CoI and external food advocacy alliance organisation.

*“Focused on getting political and senior management (service manager/ service director/DPH) buy-in first. Then moved on to engaging more topic-specific stakeholders e.g. communications team, web team, specific PH area leads”* (Policy Advocate, survey).

The commitment to pursuing this mechanism did create some anxiety amongst policy advocates who typically operate according to principles of transparency and wide engagement. Advocates cited an example of when stakeholders had been engaged before a full case had been made, creating resistance, but this mechanism was already driven by advice from the external advocacy alliance group. Fear was expressed about who to engage and when and some felt this mechanism, while important, slowed their progress down:

*“Confusion about who to approach first - conflicting messages.”​ (Policy Advocate, survey)*.

#### Gathering intelligence

This involved working to understand the existing local policy position, contracting arrangements, the policy change process, where responsibilities lie as well as data evidencing the need for policy change. This required a much larger amount of time than anticipated by many. Challenges occurred in acquiring information whilst not compromising the staggered stakeholder engagement approach.

“*Intelligence gathering right at the start of this project, that was the hardest thing I had to do: the challenge. Even with the local authority, where I’ve worked quite a few years and I thought I knew where to go and where it would be easy to find out who deals with contacts and clients, it was really difficult”* (PN001 I2).

#### Identifying policy champions

Identifying and securing backing from senior leaders who could act as credible and influential champions for the policy was crucial:

*“I think that helps in terms of ensuring that it doesn’t lose momentum, because it’s on people’s radars”* (PN001, I1).

*“We have some very passionate local council members – and that’s really good if they’re on your side…”* (PN002, I1).

The identification of policy champions appears to contribute to feeling supported, *“having someone like that, that you know is fully behind you, makes a huge difference”* (PN001, I1) and perceived level of influence *“at that more senior strategic level”* (PN003, I1).

Identifying and securing backing was often impeded by organisational complexity or restructuring and a lack of experience and confidence.

#### Building relationships

Building and maintaining relationships with those who had responsibility for policies, could influence policy change or provide intelligence was important. This was mediated by mutual respect and a clear case for change and appeared to be facilitated by the existing collaborative ethos or organisational culture within departments:

*“I think they’re [Public Health] naturally very collaborative, they network very easily and build those partnerships quite early on with projects, and that’s something that I’ve been encouraged to do as well, so I think that will help in the long run in terms of the outcomes of what we’re trying to achieve”* (PN001, I1).

Relationship building was not something that could be done quickly; investing time and drawing on established relationships was important,

*“I’ve spent a long time establishing those strong relationships within a number of different programmes and services”* (PN004, I1).

Challenges occurred in maintaining momentum over some time and staff changes undermined the process.

#### Reframing the issue

This involved pitching the work appropriately and convincingly for different audiences with consideration of differing priorities. This was particularly important given different ideological positions and positions held on commercial regulation and restriction. It also involved locating the work within an existing wider strategic objective as a vehicle for support;

*“we’re trying to apply a weight neutral and more compassionate approach to our work to tackle obesity”* (PN003, I1) and *“It sits really firmly within our strategic approach…looking at the whole environment”* (PN004, I1).

#### Amplifying the issue

Widely and openly making the case for change was not a mechanism that was widely adopted (though was on occasion used, particularly at the beginning of the project). Although this is a typical advocacy strategy, it was deliberatively avoided so that the strategic and staggered approach to stakeholder engagement was not compromised.

#### Increasing public will

Consulting the public and increasing public will were deemed important but were not typically undertaken during the evaluation period. This was seen as an activity for later in the advocacy process and was, again, influenced by a commitment not to compromise the strategic and staggered stakeholder approach.

### Contextual factors influencing change

Several contextual factors influencing policy change across the region were identified as important in facilitating change including; having a named policy advocate in place, external and CoI support, having aligned existing local strategies and objectives and the ideological position within the Local Authority. Some contextual factors acted specifically as barriers to advocacy including: the complexities of organisational structure and change including complex contracting arrangements, financial implications of the policy, and the lack of local evidence. The pandemic was identified as a contextual factor that acted as both a facilitator and a barrier.

#### A named, supported and resourced policy advocate is important for influencing policy change

Having a clearly identified and named local lead responsible for leading the advocacy process in each area was seen as crucial in implementing change. The skills and capabilities required to do this complex and demanding role effectively are reported elsewhere [12]. However, areas had varied and limited capacity and resources to support this role and competing priorities meant policy advocates were often unable to prioritise this work or spend as much time on it as they felt it needed. A sense among the policy advocates that they lacked experience in conducting advocacy work also impacted their ability to effect change.

#### Support of an external expert group and Community of Improvement facilitates change

The support of the external food advocacy alliance organisation was crucial in identifying strategies for work and the opportunity to draw on the experience of work undertaken elsewhere:

*“So it’s really good to have [NAME] there, to get her advice. She’s led the way in some ways and to replicate that is really what we’re looking at doing.”* (PN002 I1).

In particular, they advised how and when to engage stakeholders and the nature and amount of information to share about the policy change. The support and opportunity to problem solve and strategise within the CoI was also a key contextual factor in ensuring Policy Advocates were able to take work forward locally. In addition, the CoI provided a structure to maintain the prioritisation of work and ensured that the issue was amplified across the region.

#### Pre-existing and aligned local objectives or strategies act as a facilitator to change

Having local objectives or an existing strategy around obesity facilitates policy change around HFSS, offering a platform to secure political will and enabling the framing of the policy change as integral to the existing commitments of the area:

*“where it has worked better is where they’ve had the Local Authority declaration on healthy weight… they’ve [Local Authorities] found building their local relationships a lot easier” (PN007*).

*“they can see the strategic position of it with the governance that’s already in place… it’s not just coming in as an extra kind of request”* (PN004, I2).

#### Organisational and system complexity, change and unfamiliarity act as a barrier to change

Policy advocates found navigating the complex organisational system required them to both understand and identify stakeholders and key decision-makers. Importantly, understanding the current contracting arrangements and processes for change is a challenge. The more complex the organisational structure in place, the greater the barriers created.

*“So we started to look at that but it is quite complicated because of the two tiers. So we really need to understand who holds the contracts and what advertising space is where… we’ve got to work on those and make sure that we’re connecting as district and county councils in our different roles”* (PN004 I1).

This was also enhanced in areas where local government re-organisation was underway or was planned, causing uncertainty around processes and a pause in decision-making:

#### Financial concerns act as a barrier to change

Issues around financial resources were seen as negatively impacting policy change and were identified as a potential source of conflict if not managed sensitively, *“I think that’s where we need to be a little bit careful and understand what income’s generated through advertising”* (PN004, I1) and *“if they think that by introducing a policy that is restrictive it’ll impact on any income they get from marketing, that’s where it could be a bit of a conflict”* (PN002, I2). This is again a characteristic of the commercially sensitive nature of this specific area of policy change. The COVID-19 pandemic put further strain on financial resources, *“the council’s direction, especially coming out of the pandemic, economic recovery is a big factor and a localised issue for us”* (PN001, I2). Beyond these council-level concerns, a perceived personal threat to job security was also identified.

*“…if those colleagues become aware of our intention regarding this work before we’ve had chance to have conversations with them about it, that very often they pull back and refuse to engage because they worry about the loss of income impacting on their employment”* (PN003, I1).

#### The lack of a ‘northern exemplar’ acts as a barrier to change

Some Policy Advocates viewed the lack of a northern example of HFSS advertising policy change and evidence of impact as a barrier to policy change. Given the devolution of policy-making in this area in the UK, this resulted in a perceived lack of locally relevant evidence to strengthen the case for such advocacy work;

*“…in an ideal world we’d have another ‘northern’… authority that’s already done a policy, …. a lot of the evidence base is ‘south’*” (PN001, I1).

The main evidence base to support this work emanates from London which was seen to be very different in terms of demographics, culture and governance structures:

*“she [stakeholder] was very quick to point out to me, and rightly so, that that’s London, that we are different, you know, we can’t really apply the London model to us”* (PN003, I1).

#### Ideological positions within a local authority influence change

The ideological and political positioning of a local authority and the associated ‘*direction’* that led to it, as well as the potential for that to change at elections, was, to some extent identified as a risk factor for the advocacy work:

*“you know how it works, so if a Labour Party councillor supports something then the Conservative Party councillors are likely to oppose it. So having political leadership has its benefits, but the opposite can often be the case as well”* (PN003, I1) and *“When we get to Cabinet, it’s more political, so that might be a tougher kind of sell”* (PN002, I1).

Factors such as local government reorganisation were associated with a more *‘politically sensitive’* environment. In accordance, consistency appears to be beneficial for the advocacy work:

*“the great thing is our portfolio holder didn’t change in the elections either so we’ve still got his backing”* (PN001, I2).

#### Operating within a pandemic context acts as both a barrier and facilitator to change

Some Policy Advocates identified the COVID-19 pandemic as a barrier to advocacy work due to capacity issues and subsequent delays especially in the early stages of the data collection period, *“With COVID, everything has gone on hold. And Public Health, as you can imagine, has been involved in the COVID response”* (PN002, I2) and “*At the beginning of COVID I allocated very little time to it because we just couldn’t*” (PN003, I1). Others were less affected in terms of capacity but acknowledged the overall impact on Local Authorities:

“*I do COVID work as well in between it all, whereas I know that other Local Authorities have just been swamped and haven’t got the resource*” (PN001, I1).

However, the context of the pandemic was also seen as offering some unexpected opportunities for advocacy work. The pandemic raised the profile and awareness of public health and facilitated connections between different teams in the local authority through the COVID-19 response work which resulted in *“real opportunity…we’ve made some links with people that we, perhaps, didn’t directly work with before”*, this has enabled *“informal conversations”* to *“find out some information before making it more of a formal process”* (PN004, I1).

Increased public awareness of risk factors for COVID-19 was also seen as a potential facilitator of future public will and support:

*“it [the COVID-19 pandemic] has raised people’s awareness of how you are more likely to get ill if you’re from a certain background or you live in a certain area.”* (PN001, I1) and *“it’s also raised the profile of obesity, the fact that people are more adversely affected if they are obese”* (PN003, I1).

The contextual factors identified by the CoI as being specific to the four case study sites are represented in Table 2.

**Table 2:** Contextual factors associated with case study sites

**Case site 4:**​ Low level organisational complexity (Met Borough)​ Labour controlled​ Pitched within compassionate approach to all policies​ Dedicated graduate trainee to action (up to 0.5 day a week)​ Not advanced at beginning of evaluation period​	**Case site 6:**​ Low level organisational complexity (Met Borough)​ Labour controlled​ Pitched within whole systems approach to childhood obesity​ Senior experienced lead but with little capacity and no opportunity for delegating​ Work begun prior to evaluation period​
**Case site 7**:​ Low level structural complexity – Unitary Authority​ Labour controlled​ Framed in terms of healthy behaviours for obesity but moving to compassionate approach​ Policy Advocate has changed (up to 0.5 days per week)​ Work begun prior to evaluation period	**Case site 11**:​ High level organisational complexity (two-tier authority and under structural review)​ PH structure also revised​ Conservative controlled​ Led by manager new to policy change (up to 0.5 days a week but varied)​ Not advanced at beginning of evaluation period

### CMO statements and refined programme theory

Analysis of the case study sites enables us to state that:

• Site 4 achieved an increase in political will and the creation of draft guidance by adopting a staggered stakeholder approach, identifying policy champions, gathering intelligence, amplifying and reframing the issue.

• Site 6 achieved an increase in political will and had begun work on draft guidance by adopting a staggered stakeholder approach, identifying policy champions, building relationships, drawing on professional experience and developing a unified policy solution.

• Site 7 achieved an increase in political will by adopting a staggered stakeholder approach, gathering intelligence and engaging policy champions.

• Site 11 achieved an increase in political will and produced a draft business case by adopting a staggered stakeholder approach gathering intelligence, building relationships and reframing the issues.

**Fig. 4:**
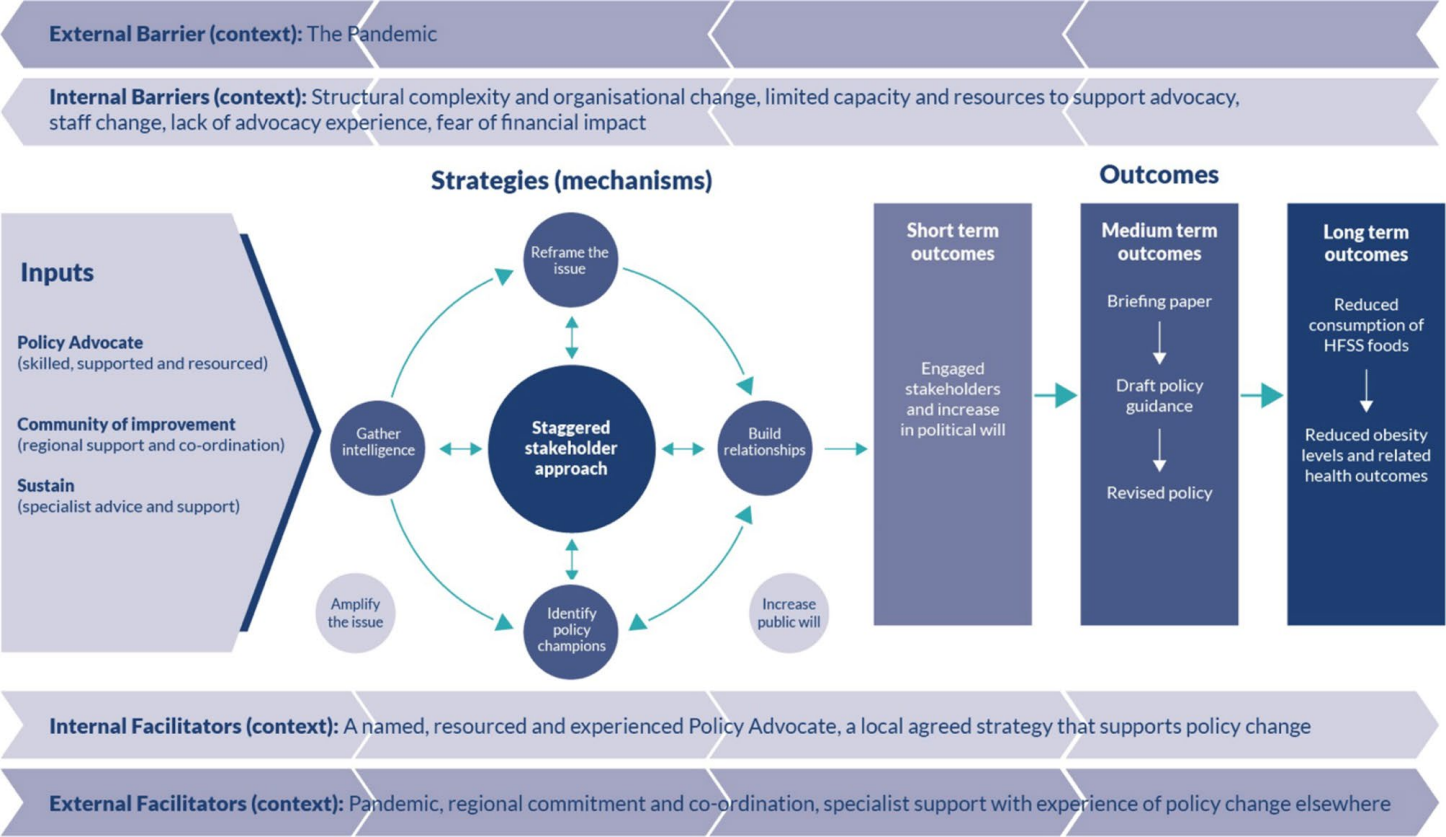
Logic model of regional advocacy process to restrict advertising of HFSS products on council-owned spaces

The revised programme theory has been represented as a logic model (see Fig. 4).

This logic model for advocacy shows the dominant short-term policy outcome is to increase the political will of influential internal policy stakeholders such as the elected members of the council, the Senior Executive Committee and senior managers within the Communications Department. To achieve this, specific inputs and the implementation of a complex set of interacting mechanisms were required. The key inputs demonstrated in this study were to have a named, skilled, supported and resourced policy advocate in each area who operated as part of a wider regional CoI that offered support and coordination alongside external advice and support from an food advocacy alliance group with existing experience in the policy field. The value of collaborating and drawing on the skills and experience of a broader set of allies such as politicians, journalists, policy groups and specialist organisations outside of public health has been identified elsewhere as an important input [36] but one which is not frequently pursued. With these in place, the locally driven mechanisms in this study revolved around a dominant central mechanism described as a strategic and staggered stakeholder engagement which influenced all areas of activity. This central mechanism was triggered by the unique sensitivities and fears of industry counter-lobbying surrounding this specific policy context. The mechanism of gathering intelligence, which includes understanding the local and national evidence, local contracting position and policy change process, informs decisions about subsequent mechanisms including how to build relationships with stakeholders, how best to frame the issue and who could best champion policy change. The mechanisms were typically applied in each area but to different degrees and not necessarily in the same order. They are not seen as sequential but rather as mechanisms that influence each other and which may be employed iteratively at different stages of the advocacy process. Decisions about when to engage in each mechanism were taken locally as a form of pragmatic real-time problem solving and were informed by local context and progress towards the policy goal. Mechanisms of amplifying the issue and increasing public will, which are typical in advocacy work [16, 37], were not seen operating in this study but were identified as mechanisms that may be used later on in the process.

## Discussion

Evidence of the impact of reducing advertising of HFSS foods is growing [9, 10] and demonstrates its potential to contribute to public health obesity goals when implemented as part of a wider strategy. What is less well understood is how this policy change can be achieved, particularly when pursued across a UK region outside London. A process evaluation of policy change in London highlighted important lessons for implementation but reflected an experience in a very specific governance context [38]. The restriction of HFSS advertising may present particular commercial and ideological sensitivities and existing public health advocacy frameworks designed to support practitioners in their role as policy advocates may lack relevance in this particular policy realm because of this as well as becuase advocates are working at a local level and trying to effect change within their own organisation. This study is important in offering insight into the different mechanisms employed in the advocacy process of this sensitive area of the policy change and identifies the associated challenges and opportunities that they offer, as well as the important contextual factors that require consideration when pursuing policy change in this area.

Findings from this paper show that implementing policy change in local HFSS advertising is time-consuming and requires an extended time period for implementation. In areas with more complex governance arrangements or areas facing organisational restructure or political change, the process takes longer still. During the 12 months of this evaluation, no policies were fully implemented. However, considerable work was undertaken across the region and progress was made in understanding the local policy positions and contractual situations, developing draft guidelines ready for implementation and securing the political will of key internal policy stakeholders, all of which constitute important foundational work for policy change. The length of time required for public health advocacy of this type is documented elsewhere with calls to recognise the smaller policy wins such as those described above when evaluating advocacy work [39]. If they are to be successful, local authorities considering similar policy changes should be prepared to support and resource an advocacy process that may take many months or indeed years. In accordance, a case has been made for increasing public sector resources for advocacy work if it is to compete with the substantial financial backing of corporate lobbyists [36].

Many of the documented examples of public health advocacy work, such as in gambling and alcohol consumption, describe the use of similar mechanisms as those reflected in Fig. 4 and face similar challenges, especially those operating in a context linked to big national or multinational commercial interests but looking to make a change at a local level [40, 41] Other public health advocacy frameworks have been presented in the literature to support public health practitioners in this role and these demonstrate overlap with some mechanisms identified in this study. The framework for nutrition advocacy developed by Cullerton et al [16] and discussed above, shows a similar focus on gathering intelligence including of evidence, reframing the issue, building relationships and identifying policy champions. Similarly, a public health advocacy framework developed in the policy realm of gambling [39] also overlaps in its recommendations for strategies of securing evidence, leadership support and careful framing of the issue. In addition, mechanisms identified by Cohen et al [37] similarly include strategic framing of the issue, gathering information and building collaborations.

Where the logic model developed from the findings in this study differs from existing advocacy frameworks is around the use of mechanisms to amplify the issue, increase public will and the processes for building stakeholder engagement and collaboration. Cullerton’s framework recommends that policy advocates ensure their cause is clear and continually heard, both by policymakers and the public and that gaining public support and demonstrating this to policy makers was crucial for influencing policy change. Similarly, two of David et al’s [39] eight-step framework are based on similar principles: communicate the vision and empower others to act on the vision. These were mechanisms that were deliberately avoided by the policy advocates in this study due to fears about managing resistance and counter-lobbying, both internally and externally to the organisation, and this perhaps reflects the local and internal advocacy context here rather than the national advocacy seeking to influence change from an external position that is the focus of Cullertons’ study. These fears, which were strongly held by policy advocates in this study, related to potential resistance caused by financial impact concerns, an ideological reluctance of stakeholders to be seen as a ‘nanny state’ and fears about the power of the commercial sector and counter lobbying. Examples were given in the data of occasions where internal stakeholders were informed of policy goals before a solid case had been formed and were consequently resistant. These fears are also echoed in the advocacy literature [37, 39, 42] and form part of the ‘corporate playbook’ to protect business interests [36]. They are illustrative of the powerful influence of the commercial determinants of health whereby the food industry may exert influence through marketing, lobbying, extensive supply chains which amplify company influence and corporate citizenship strategies which deflect attention. These corporate strategies influence the availability, cultural desirability and prices of unhealthy food and shape the environment and choices of individuals and communities [43] and the power large food industries holds has been identified as the dominant cause of ‘policy inertia’ across governments [44] and can be seen to drive the cautious approach underpinning the work of policy advocates in this study. Fears that industry may counter-lobby this specific policy change are well founded with research undertaken after the policy change across Transport for London showing that there was substantial opposition from food and advertising industry actors. They employed strategies such as exaggerating the costs of the change, attempting to discredit evidence, and raising potential of legal actions. They were shown to have significant access to policy processes and the resources to simplify their presence [45] While supported by the majority of policy advocates involved in this project, the avoidance of wide engagement and amplification of the issue does present challenges. For example, this slowed down the process and it impacted the ability to fully implement other mechanisms such as the gathering of local information about contracts and policy processes.

The decision not to build wide networks as part of this advocacy process runs counter to established policy network thinking where a well-connected policy advocate who has built a strong and wide network gains greater access to information and political influence and therefore power [15]. One of the factors, for example, that facilitated the effective advocacy for tobacco control policies in Scotland was the establishment of a wide formal network of individuals and organisations including those not necessarily expected to have an interest in tobacco control [46]. However, as Cullerton goes on to explore, the nature of those connections may prove to be more important than the number of connections [15]. In line with this, results in the current study do show that while policy networks here are small, the political support of those that have been actively engaged has been secured. The need to secure the support of a senior policy champion who understands and supports the policy goal and can mediate for the policy advocate is fundamental. Typically, policy advocates chose senior public health staff such as the Director or Assistant Director of Public Health or Local Authority elected members who hold portfolios for health. The informed and senior position of these policy champions made them well-placed to give authority to the policy advocate, speak on their behalf, provide access to decision-makers and use their strategic position to improve the effectiveness of policy advocates. The relationship between the policy advocate and their champions was key. In their analysis of policy networks, Cullerton et al [15] described the role of the policy champion as acting as a broker for the policy, providing mentorship to policy advocates, acting as an aggregator and conduit of information and intervening in the advocating process to mediate trust. Whilst building a policy network slowly may be important in this sensitive policy area so is the strategic identification of a policy champion early on in the advocacy process given that this may compensate in some ways for the limited network in place. In a regional approach where policy advocates are operating across different Local Authority settings, connecting the policy champions as a coalition across the region may be a useful additional strategy for maximising their input [36].

The parallel decision not to engage the public or any stakeholders outside the Local Authority runs counter to Public Health principles of transparency and openness [47] and sat uncomfortably with some of the policy advocates interviewed in this study, despite their commitment to it as an approach. However, paternalistic public health advocacy of this type is not uncommon. Carlisle’s framework for advocacy shows two intersecting axes which move from facilitation (with communities) to representational (on behalf of communities) and from cases (individual lifestyle and behaviour) to causes (social policy and structure) [41]. This example of advocacy sits in the representational/causes domain and although it may be seen as ‘top down’ it is seen as justified when seeking to address inequalities in health through social transformation and challenging the vested interests behind the commercial determinants of health. In this way, it reflects ‘top down’ advocacy for tobacco control or taxation on sugary drinks [46, 48]. However, a process evaluation of implementing similar advertising restrictions across the Transport for London network showed that early consultation and close communication with both industry and the public were important facilitating factors in developing the policy [38]. While the policy change is limited to restricting advertising on council-owned spaces where the internal policy actors may be resistant but are not adversarial, the discreet approach might be manageable. However, to extend the policy, for example onto transport networks or other commercially controlled spaces where communication with private sector actors will be necessary, the advocacy process will need to leverage public support and do so carefully. This should draw on the lessons from similar campaigns to gain public support and pre-empt industry resistance, such as during the implementation of taxes on sugar-sweetened beverages both in the UK and internationally [49–51] and should include efforts to counter corporate arguments around choice, personal responsibility and freedom by presenting an alternate set of values centred on social equity with, what Lacy-Nichols et al. [36] describe as ‘*continual re-emphasise that the ultimate causes of poor health and health inequity are structural’* [Pe1070].

A further distinction between the logic model produced in this study and existing advocacy frameworks is the attention given to context and the data here demonstrate the importance of identifying and navigating the local context during the advocacy process. Presenting the evidence of effectiveness, for example, was seen as a key driver to secure political will but importantly needed local relevance. Evidence from the same policy change in London was seen as an important tool, however, to ensure that this was not dismissed as locally irrelevant it needed to be situated alongside local epidemiological and demographic data and used within a narrative that recognised the local issues. In particular, the localising of the evidence and the policy message needs to position the policy change as part of, rather than additional to, existing local objectives and commitments, rather than in addition to. Capitalising on existing commitments such as to the Healthy Weight Declaration or a local strategic objective and crafting narratives tailored to the local ideological position and contracting arrangements were seen as an important ways of framing the issue. This reflects the tension that exists in advocacy between the role of evidence and the influence of power, politics, values and ideology [37].

Finally, the importance of the collective approach to policy change across a region, with each local authority working individually according to their own context, but towards the same goal and with collective support, was highly valued. The synergy created by this approach is important. However, many of the barriers faced at a local level that impacted the time taken to effect change, might be more efficiently addressed if this regional approach were accompanied by organised public health efforts at a national level. In their ‘public health play-book’, Lacy-Nichols et al. [34] identify the strategies adopted by commercial actors and offer a set of strategies for public health to counter powerful commercial influences that impact health and well-being of populations. These eight strategies include recommendations for building and protecting public health practitioners as advocates as well as strategies for collaborative working and challenging, exposing and dividing commercial interests. While these offer relevance to advocacy at a local level, their impact and effectiveness are likely to be far greater if pursued and championed nationally.

## Conclusion

In undertaking a realist evaluation of a regional approach to the restriction of advertising of HFSS products on council-owned spaces, the findings demonstrate the extended period required to effect change, the importance of valuing short-term policy outcomes, such as increasing political will, and the importance of a resourced policy advocate supported by a Community of Improvement in leading this work. The resultant logic model demonstrates the complex interplay of mechanisms used which were dominated by a strategically staggered approach to stakeholder engagement. While there is overlap in the advocacy processes used in this study with existing public health advocacy frameworks, the particularly commercially sensitive nature of this policy change and the specific regional context means their relevance has limitations. Supporting and resourcing public health practitioners through the complex and time-consuming process of advocacy, is crucial if local public health teams are to ensure the wider determinants of health, and particularly the commercial determinants of health are to be addressed.

## Data Availability

The complete data set collected through this study will not be made publicly available as it cannot be fully anonymised. However, upon reasonable request anonymised data can be provided by contacting Professor Susie Sykes sykess@lsbu.ac.uk. Materials used in this study are available: https://osf.io/s7bvm/ DOI 10.17605/OSF.IO/S7BVM.

## Abbreviations

HFSS products: products that are high in fat, salt and sugar
CoI: Community of Improvement
